# Editorial: The role of viruses in marine environments

**DOI:** 10.3389/fmicb.2024.1437050

**Published:** 2024-06-06

**Authors:** Min Jin, Yi Gong, Claire Geslin, Tianliang He, Xiaobo Zhang

**Affiliations:** ^1^State Key Laboratory Breeding Base of Marine Genetic Resource and Southern Marine Science and Engineering Guangdong Laboratory (Zhuhai), Third Institute of Oceanography, Ministry of Natural Resources, Xiamen, China; ^2^School of Life Sciences, Nanchang University, Nanchang, China; ^3^Laboratoire de Biologie et Ecologie des Ecosystèmes marins Profonds (BEEP), UMR 6197 (UBO-CNRS-Ifremer), Plouzané, France; ^4^College of Animal Science and Technology, Anhui Agricultural University, Hefei, China; ^5^Laboratory for Marine Biology and Biotechnology of Pilot National Laboratory for Marine Science and Technology (Qingdao) and Southern Marine Science and Engineering Guangdong Laboratory (Zhuhai), College of Life Sciences, Zhejiang University, Hangzhou, China

**Keywords:** marine viruses, phages, viral diversity, viral ecology, virus-host interaction

Viruses are the most abundant biological entities in the ocean. They are major players in marine ecosystems, as they control host abundance, influence host diversity and evolution, and affect local and global biogeochemical cycles. In the last decades, research on marine viruses has emerged as a significant and distinct field in marine biology and ecology. The development of advanced metagenomic sequencing methods, analysis pipelines, and tools has led to an increased understanding of the diversity and ecology of viruses in the marine ecosystem. However, there are still unexplored areas of research on viruses in marine environments. The goal of this Research Topic is to provide a forum to advance the study of marine viruses. The Research Topic features eight noteworthy papers focusing on different aspects of marine viruses, including technological optimization for visualizing marine viruses, isolation and characterization of novel marine phages, host-virus interaction in marine bacteria, crustacean and jellyfish models, and ecology of cyanophages and bathypelagic viruses.

Scanning electron microscopy and scanning transmission electron microscopy are important approaches to visualize and image viruses and virus-like particles (VLPs). Kakol et al. developed and tested three different protocols for sample preparation and imaging. They found that these three different protocols have distinct advantages and disadvantages for different viral samples, highlighting that the choice of the analytical procedure significantly influences the resolution and preservation state of the observed phages and VLPs. To this end, the authors suggested that the appropriate imaging technique should be carefully selected based on the specific objectives of the project and the nature of the samples being investigated to obtain the best images of the viruses.

In the era of metagenomics, it remains crucial to isolate new viruses and to characterize the interactions between virus-host model systems. Wang et al. isolated and characterized a novel T4-like cyanophage *Nanhaivirus ms29* from the ocean basin in the South China Sea. Bioinformatic analysis showed that this cyanophage is mainly distributed in temperate and tropical epipelagic waters. Abundant auxiliary metabolic genes were identified in the phage genome, possibly reflecting a genomic adaption to the oligotrophic environment. Further phylogenetic analysis demonstrated that *Nanhaivirus ms29* is distinct from other known T4-like cyanophages and belongs to a novel genus within the family *Kyanoviridae*.

Liu et al. isolated and characterized another novel estuary cyanophage S-CREM2, which represents a novel viral genus belonging to a newly proposed T4-like cyanophage clade named cluster C. A prominent feature of cyanophage S-CREM2 is that it possesses the longest tail (~418 nm) among isolated cyanomyoviruses and encodes six tail-related proteins that are exclusively homologous to those predicted in the cluster C cyanophages. Moreover, S-CREM2 virion may carry three regulatory proteins, which may play a crucial role in phage replication at the initial stage of infection. The studies carried out by Wang et al. and Liu et al. offered valuable insights into the diversity and phylogeny of marine cyanophages.

Viruses and hosts are in an ongoing evolutionary “arms race.” To survive the constant infection of viruses, hosts have evolved mechanistically diverse defense strategies. Zeng et al. explored the evolutionary adaption of *Vibrio parahaemolyticus* against two kinds of marine phages. They found that *V*. *parahaemolyticus* inhibited phage adsorption to its surface by mutating the host flaG gene. However, this anti-phage strategy also led to the reduced growth competitiveness of the anti-phage mutant strain. These results suggested that selection pressure on different anti-phage strategies depends on the trade-off between mortality imposed by phages and the fitness cost of the defense strategy under the given environmental conditions.

Focusing on the antiviral response in marine crustaceans, Hu et al. evaluated the role of apoptosis inhibitor 5 (API5) in the immune response of mud crab (*Scylla paramamosain*) against White Spot Syndrome Virus (WSSV) infection. The authors found that API5 was upregulated upon WSSV infection, and the silencing of API5 led to increased WSSV copy numbers and apoptotic rate of hemocytes, highlighting its important role in the immune response. Further investigation showed that API5 interacted with Heat Shock Protein 20 (Hsp20), which could promote cell apoptosis of hemocytes and reduce viral copy numbers.

Focusing on the role of phage-bacteria interaction in metaorganism biology, Stante et al. exposed moon jellyfish *Aurelia aurita* to individual phages and a phage cocktail, and monitored polyp survival and morphology, as well as microbiome changes. The results showed that phage exposure altered the microbiota associated with *A. aurita*, thereby leading to recoverable malformations in polyps without affecting their survival. Moreover, this study also demonstrates the overall resilience of the *A. aurita* metaorganism facing phage challenges, since the main colonizer of *A. aurita*, likely a novel *Mycoplasma* species, showed resilience upon phage exposure.

Over the past decades, the application of metagenomics has greatly expanded our knowledge of marine viruses. However, metagenomic studies on bathypelagic viruses remain limited. Sun et al. analyzed the 16S rRNA sequencing and viral metagenomic sequencing data of 25 samples collected from five different bathypelagic ecosystems. The low phage/host ratios in these abyssal ecosystems were different from shallow ecosystems, indicating the prevalence of lysogeny among bathypelagic viruses. Moreover, the correlation analysis revealed several phage–bacteria interaction networks of potential ecological relevance, providing novel insights into the interactions between bathypelagic bacteria and their phages.

Lastly, viral lysis of host cells releases organic matter and nutrients that affect the surrounding microbial community. Man et al. explored how viral lysis of *Synechococcus* affected the co-existing bacteria and nutrients in the culture. The bacterial community structure was altered after cyanophage infection, and increased bacterial diversity and richness were observed. The nutrients in the cyanophage-added culture featured decreased nitrate and increased ammonium and phosphate, which is coupled with the viral progeny production and increased substance transport and metabolism potentials of the bacterial community. Furthermore, cyanophage infections contributed to the bacterial production of methane-related compounds and refractory organic matter in the culture. This study helps to deepen our understanding of the impact of viral lysis of cyanobacteria on the surrounding marine microbial community.

In conclusion, the studies presented in this Research Topic provide valuable insights into the diversity and ecology of marine viruses. However, there is still much to be learned about marine viruses, including the diversity and ecology of marine ssDNA and RNA viruses, the diversity and ecology of deep-sea viruses, novel mechanisms of virus-host interactions, and the role of marine viruses in local and global biogeochemical cycles. The further development of molecular tools based on the cultivation and characterization of novel marine virus-host models is needed to address these gaps in our knowledge. Moreover, culture-independent technologies, such as innovative bioinformatic tools that efficiently interpret enormous viral metagenomic sequences and experimental procedures that link viruses and their hosts directly in natural samples, are also required to elucidate the critical roles viruses play in marine ecosystems.

## Author contributions

MJ: Writing – original draft. YG: Writing – review & editing. CG: Writing – review & editing. TH: Writing – review & editing. XZ: Writing – review & editing.

